# Medical Opinion and Movement

**Published:** 1907-07-13

**Authors:** 


### Medical Opinion and Movement.

A little time back we referred in these columns to the debate which took place at the Congress of Medical Practitioners in Paris this year on the question of doubling the rates of charges for medical attendance on Sundays and holidays, in order to secure more certain rest for medical men from their labours on these days and put a check upon needless calls for their services. We understand now that the Medical Syndicate for the Department of the Rhone has already given practical effect to the resolution passed by the Congress in favour of such a measure, and has issued a notification to the public that from July 1 fees for visits and consultations on Sundays and holidays will be fixed at double the usual rate.

Among those who have been the recipients of honours on the occasion of the celebration of the King's birthday there are 14 medical men, 10 of whom are members of the naval or military medical services. The honour of knighthood has been conferred upon Mr. Henry Rosborough Swanzy, F.R.C.S. Irel., and Mr. Alderman Thomas Boor Crosby, M.D., F.R.C.S.Eng. Sir Henry Swanzy is President of the Royal College of Surgeons in Ireland, and late President of the Ophthalmological Society of the United Kingdom. He is surgeon to the Royal Victoria Eye and Ear Hospital, Dublin, and also to the Adelaide Hospital, Dublin. His contributions to the literature of ophthalmology are numerous, and he is a well-known authority in that branch of the profession. Sir Thomas Crosby is a Sheriff and Justice of the Peace for the City of London. Sir William McGregor, K.C.M.G., C.B., Governor and Commander-in-Chief of Newfoundland, has been promoted to a Grand Commandership of the Order of St. Michael and St. George. He graduated M.D. at Aberdeen, and held resident appointments at the Glasgow Royal Infirmary and at the Royal Lunatic Asylum, Aberdeen. He then became assistant medical officer in the Seychelles and chief medical officer in Fiji, and afterwards held many distinguished colonial official positions. Professor Edwin Ray Lankester, F.R.S., Director of the Natural History Museum, who has been made a Knight Commander of the Bath in recognition of his services in the interests of science, though not a medical man, also deserves mention in these columns.

The mercurial treatment of syphilis by intramuscular injections, either of metallic mercury or of its salts, has become much more extensively used in recent years, owing largely to the improvements in technique, by which previous troubles, such as painful nodosities, abscesses, and embolism, have been to a great extent avoided. The method has been especially advocated and adopted by French syphilologists, and it has been clearly demonstrated that patients can be brought under the influence of the drug with greater rapidity and certainty than by any other method. The soluble and the insoluble salts, as well as metallic mercury in suspension (*l'huile grise*) have been employed for the purpose, but it is generally admitted that the soluble salts are not so efficacious, and the injections must be repeated at frequent intervals. Calomel and metallic mercury are by far the most energetic forms of the drug, but their use is accompanied by a greater or less degree of pain. The metallic mercury causes much less inconvenience, but its suspension in a suitable vehicle for injection has been a question of some difficulty. Colonel F. J. Lambkin, of the Military Hospital, Rochester Row, claims to have solved these difficulties by using palmitin as a base and adding as an analgesic equal parts of absolute creosote and camphoric acid. The mercurial cream thus formed should contain 10 per cent. of mercury and 20 per cent. camphorated creosote. The calomel preparation is made up only half that strength. We understand that these creams can be obtained from Messrs. Oppenheimer in aseptules containing each a maximum dose of 15 minims.

Medical men have always been ready to offer their services wherever they were needed, regardless of compensation when adequate remuneration was not forthcoming. In this way has arisen all the gratuitous work carried out by the profession at hospitals and medical institutions, and the medical attendance in connection with dispensaries and friendly societies at unremunerative rates of compensation. All these things have been arranged largely in the name of charity, but the profession is now exposed to the danger of being taken at their own valuation; and the actual commercial value of their services estimated on the same low scale. The recent action of the Westminster Borough Council in regard to the provision for the medical attendance of their employés is a case in point. Hitherto a workman, in case of illness or accident, has been required to furnish a medical certificate weekly from a district medical officer appointed by the Council in order to obtain his sick pay. The cost of these certificates (2s. 6d. each) has been borne by the Council, and has amounted to between £200 and £300 per annum. A joint sub-committee of the Works and Highways Committee, appointed to determine the best means of meeting the Council's responsibilities under the Workmen's Compensation Act, reports that the average sum paid by friendly societies for medical attendance, medicine, and certificates amounts to 4s. per man per annum. It is proposed, therefore, that this would be an adequate rate of payment for the Council's workmen within a three miles' radius of the City Hall, and that a further 2s. per head should be paid for those beyond this radius. In this way it is reckoned that their 1,000 employés will be provided for at a cost of £225 per annum. In other words, the ratepayers' money is to be spared in this instance by exploiting the doctor. Such a scheme is not only unfair to the doctor, but also to the employé. No medical man can possibly give adequate time and attention to his patients on such a low scale of remuneration. In this case there is not even the plea of charity. It is a purely commercial transaction, and we can only hope that the Council will not succeed in obtaining the services of the profession on such a basis.

